# Nodular Fasciitis of the Face Following an Insect Bite: A Case Report

**DOI:** 10.7759/cureus.97767

**Published:** 2025-11-25

**Authors:** Duaa M Gafar Ali, Ahmed E Musa, Sahar K Elhagahmed, Eelaf S Osman, Abdelazim Mohammed, Mohamed Shawgi

**Affiliations:** 1 Trauma and Orthopedics, South Tees Hospitals NHS Foundation Trust, Middlesbrough, GBR; 2 Family Medicine, Swansea Bay University Health Board, Swansea, GBR; 3 Hematology, South Tees Hospitals NHS Foundation Trust, Middlesbrough, GBR; 4 Acute Medicine, South Tees Hospitals NHS Foundation Trust, Middlesbrough, GBR; 5 Radiology, South Tees Hospitals NHS Foundation Trust, Middlesbrough, GBR; 6 Radiology, James Cook University Hospital, Middlesbrough, GBR

**Keywords:** benign tumor, nodular fasciitis, pseudosarcomatous fasciitis, pseudosarcomatous fibromatosis, temple lesion

## Abstract

Nodular fasciitis (NF) is a rare, benign proliferation of fibroblasts that can mimic soft tissue sarcomas. It most commonly occurs in the upper extremities and trunk, while head and neck involvement in adults is uncommon. We report a case of a 50-year-old female presenting with a progressively enlarging right temporal lesion following an insect bite. Imaging revealed a well-circumscribed, low- to iso-dense lesion based in the temporal fascia with mild fluorodeoxyglucose (FDG) uptake on positron emission tomography-computed tomography (PET-CT), consistent with benign pathology. Histopathological examination demonstrated spindle-shaped myofibroblasts with a mixed inflammatory infiltrate and no evidence of malignancy. Immunohistochemistry was positive for Actin, supporting the diagnosis of NF. The lesion regressed spontaneously following corticosteroid therapy. This case highlights an unusual presentation of NF in the temporal region of an adult. Awareness of its clinical and radiologic features is essential to avoid misdiagnosis and unnecessary aggressive management.

## Introduction

Nodular fasciitis (NF) is a rare, benign lesion of unclear aetiology consisting of proliferated reactive fibroblasts, usually affecting subcutaneous tissue, muscles, and fascia. It is often a self-limiting soft tissue lesion that grows rapidly, occasionally raising concern for malignancy on initial assessment. It was first known as subcutaneous pseudosarcomatous fibromatosis as it was described by Konwaler et al. in 1955 [1]. The primary etiology is not clear, probably related to a reactive process to minor trauma, especially with cases of blood vessels radiating from the center of the lesion with residual fibrin clots [2]. However, recent literature links NF to the Ubiquitin-specific protease 6 gene (USP6)-associated neoplasia [3]. In our case, there was a history of an insect bite. Because it presents as a solitary tumor and it tends to grow rapidly over a short period of time, it could be misdiagnosed as a malignant soft tissue tumor (e.g., sarcoma), especially given its high cellularity, high mitotic index, and its infiltrative borders on histopathology [4].

We present a case of an unusual site for NF in adults and discuss the radiological, histological, and immunohistochemical findings. We aim to raise awareness of this rare, benign lesion, as misdiagnosing these types of lesions as malignant tumours could lead to overtreatment, primarily when they manifest in unusual sites like the head and neck.

## Case presentation

**Figure 1:**
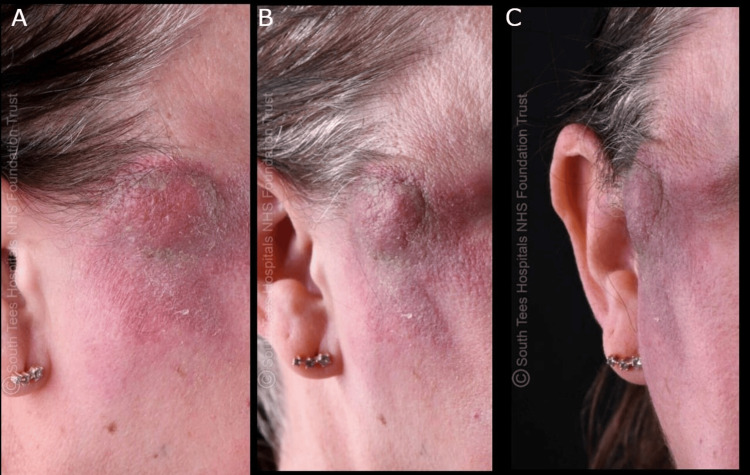
(A-C) Images demonstrating the extent of the lesion. The lesion appears erythematous, firm, lumpy, and slightly raised, with overlying scaling and induration, located in the right temporal region and measuring approximately 40 × 40 mm. The patient has provided written consent for her images to be published in the journal.

A 50-year-old female presented with a progressively enlarging right temple lesion that began after an insect bite three months prior (Figure 1). The swelling was associated with discomfort and right eye puffiness, but there was no change in vision. On assessment, there was mild localized tenderness around the insect bite area. The lesion did not respond to oral flucloxacillin, prompting referral to a tertiary hospital for suspected right-sided facial abscess. On hospital assessment, the swelling was firm, mildly tender, erythematous, lumpy, and measuring approximately 40 x 40 mm. There was no visual loss or other ocular signs. Doxycycline 200 mg once daily for one week was prescribed with no clinical improvement. Blood tests, including complete blood count (CBC), C-reactive protein (CRP), erythrocyte sedimentation rate (ESR), and human melanoma black 45 (HMB-45) (melanoma marker), were unremarkable. On follow-up, the lesion appeared slightly pigmented and red, with an irregular surface, surrounding inflammation, and a prominent, smooth, regular, non-tender lymph node beneath the angle of the jaw.

**Figure 2:**
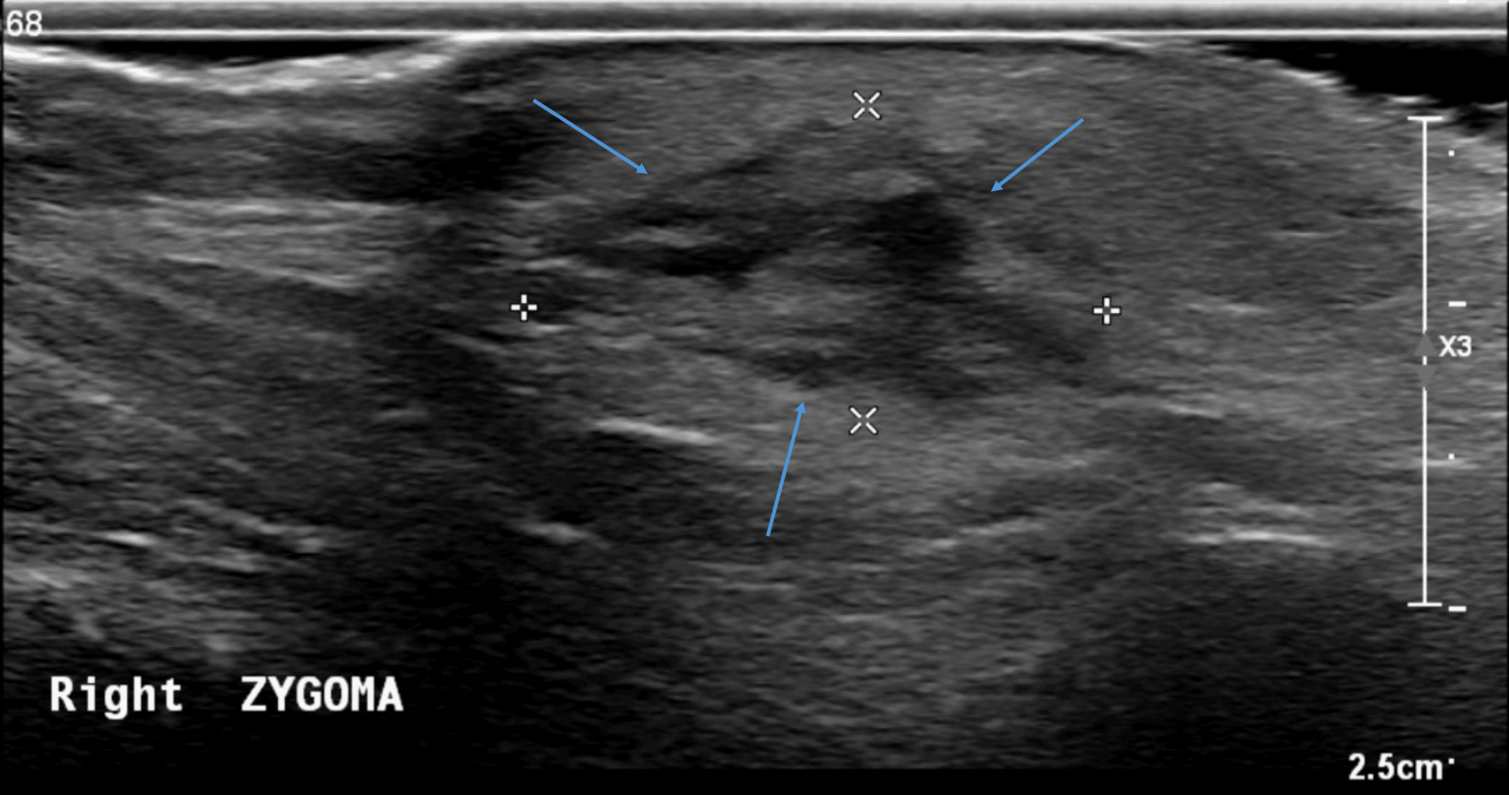
Ultrasonography of the lesion (May 2025). There is predominantly hypoechoic oval-shaped deep subcutaneous lesion against the right zygomatic arch (arrows), measuring approximately 19 x 10 x 23 mm. The lesion appears only minimally compressible, with no evidence of a foreign body or a tract to the skin surface.

**Figure 3:**
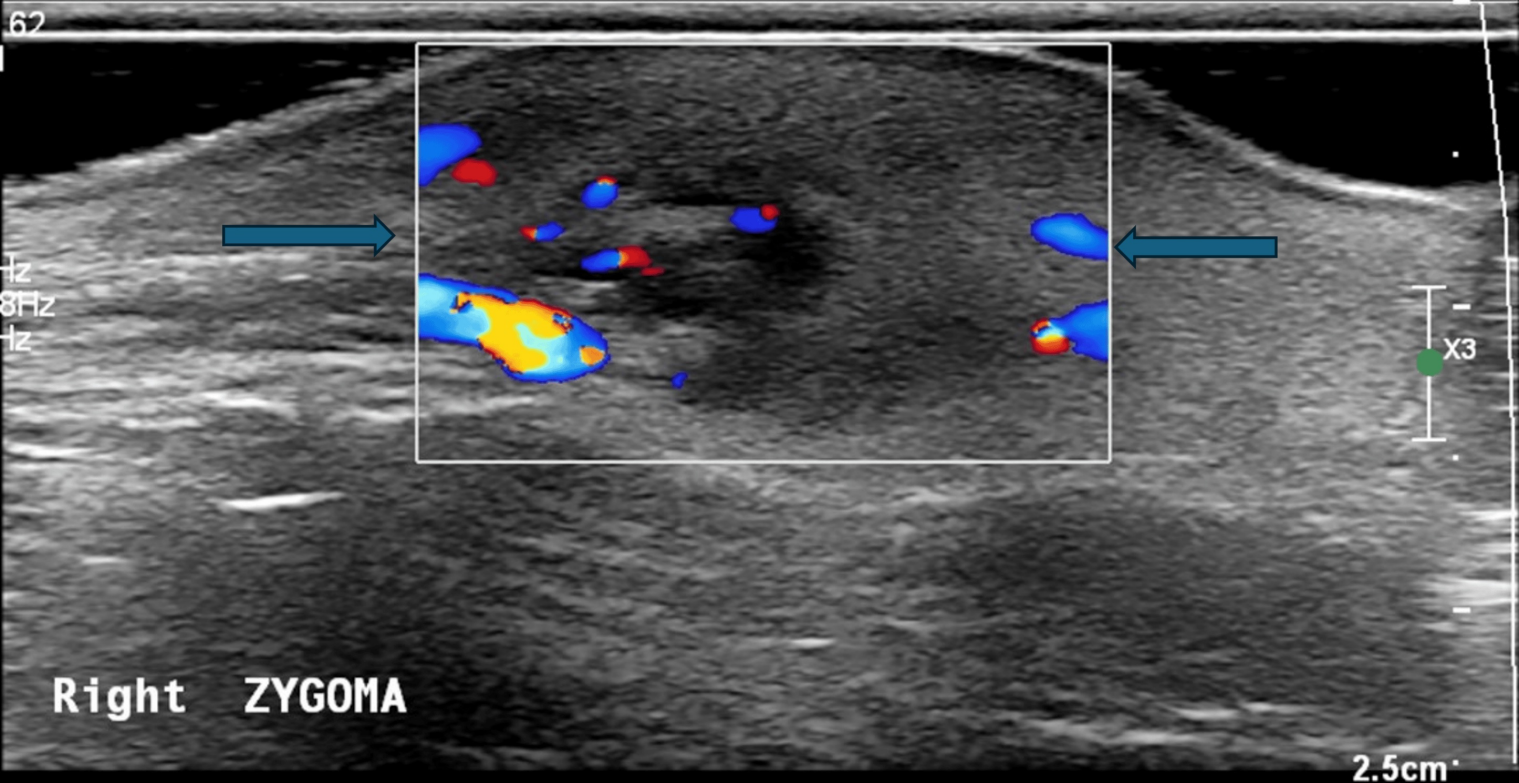
Ultrasonography of the lesion with color Doppler (May 2025) demonstrating increased vascularity within and around the lesion (arrows), which may suggest inflammatory changes.

Ultrasonography of the lesion revealed a hypoechoic, diffuse region of subcutaneous fatty tissue with hyperechoic fatty change, measuring approximately 19 × 10 × 23 mm (Figure 2). This was associated with increased vascularity and a small region of possible fat necrosis within the lesion (Figure 3).

**Figure 4:**
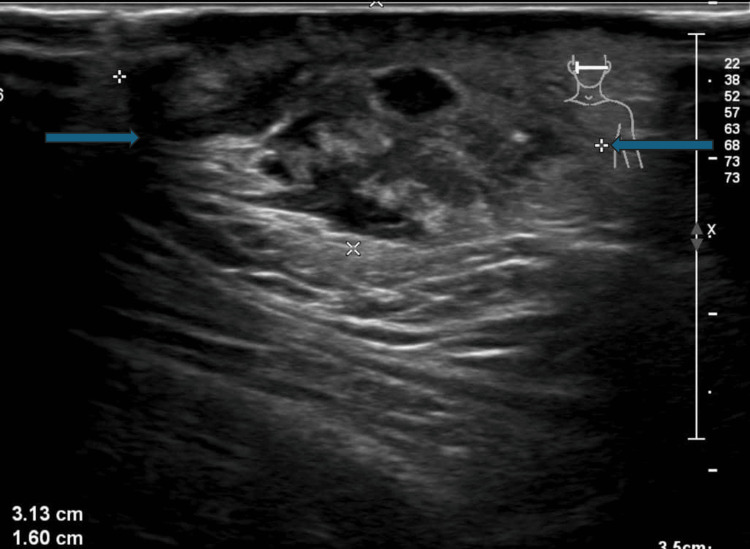
Follow-up ultrasonography of the lesion (June 2025). Ultrasound image showing a lesion with a significant interval increase in size, now measuring approximately 31 mm in maximum diameter (arrows). The lesion is located within the superficial subcutaneous tissue, in immediate contact with the overlying thickened skin and hypodermis. It appears as a heterogeneous, poorly defined complex area containing small anechoic fluid foci (largest approximately 4 mm) and hyperechoic components without posterior shadowing. Hypoechoic dermal and hypodermal tunnels are noted, suggesting dilatation of lymphatic ducts. Marked surrounding inflammatory oedema is evident.

**Figure 5:**
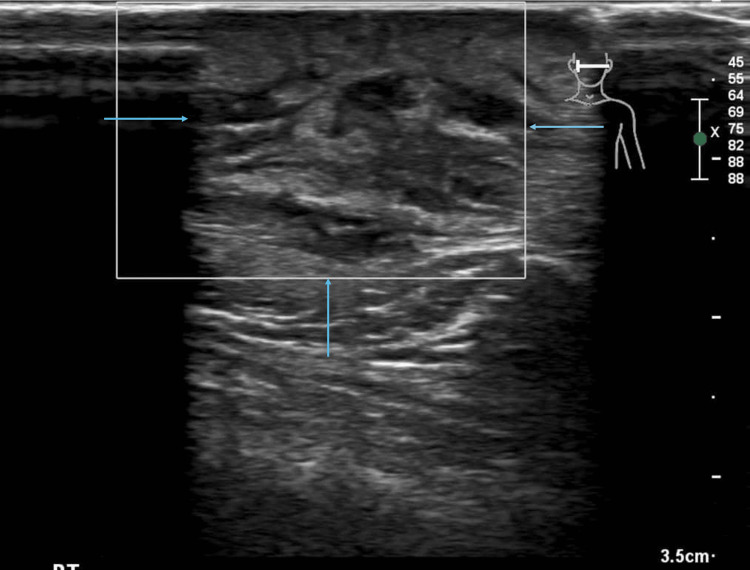
Follow-up ultrasonography of the lesion with color Doppler (June 2025) showing no significant intralesional vascularity (arrows), in contrast to the findings on the previous ultrasonography.

A follow-up ultrasound scan 19 days later revealed heterogeneous, poorly defined complex areas with some small fluid components and hyperechoic areas without posterior shadowing. Findings were associated with dilatation of lymphatic ducts, significant surrounding inflammatory oedema, marked dermal and hypodermal thickening, and a small reactive intraparotid lymph node, favoring the possibility of an infective process within the subcutaneous tissue. However, the lesion demonstrated a progressive increase in size with no clear underlying etiology identified (Figure 4). Subsequent color Doppler ultrasonography revealed an absence of significant intralesional vascularity, in contrast to the increased vascularity noted on the prior examination, indicating decreased vascular perfusion and suggesting lesion regression (Figure 5).

**Figure 6:**
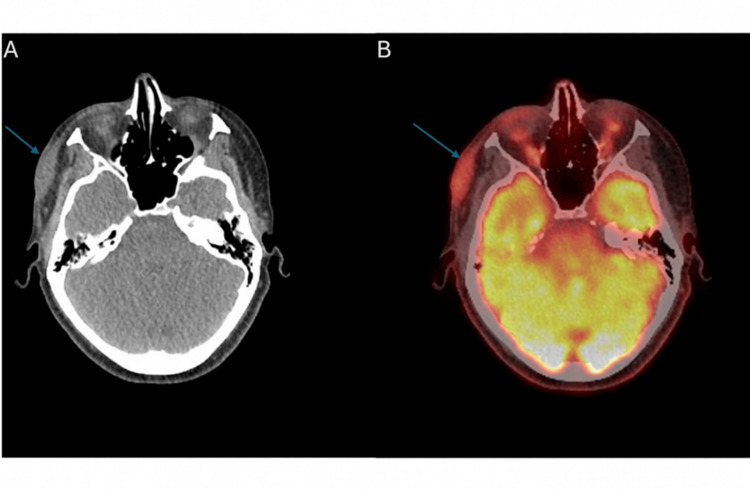
CT and PET-CT imaging. (A) Computed tomography demonstrating a 35 × 11 mm well-circumscribed, ovoid-shaped soft tissue thickening centered on the right temporal fascia just above the zygomatic arch (arrow). (B) Positron emission tomography-computed tomography (PET-CT) showing mild fluorodeoxyglucose (FDG) uptake within the same 35 × 11 mm well-circumscribed, ovoid-shaped soft tissue thickening centered on the right temporal fascia just above the zygomatic arch.

A PET scan revealed mild fluorodeoxyglucose (FDG) uptake within a 35 × 11 mm well-circumscribed, ovoid-shaped soft tissue thickening centered on the right temporal fascia, just above the zygomatic arch, with no other FDG-avid subcutaneous or cutaneous lesions or lymphadenopathy (Figure 6). Low-grade FDG uptake within a 10 mm nodule in the right parotid gland was noted, suggestive of an incidental benign parotid neoplasm. The appearances on positron emission tomography-computed tomography (PET-CT) were not suggestive of sarcoma or melanoma; however, the sensitivity of the study was reduced as the patient was on steroids.

On histopathology, there were mild epidermal changes with very prominent dermal and subcutaneous infiltrate associated with areas of a fibrosing process. The infiltrate consists of SMA-positive myofibroblasts, lymphocytes without cytological atypia, histiocytes, eosinophils, neutrophils, and numerous plasma cells. There was a population of spindle cells with mild to moderate nuclear pleomorphism infiltrating into the subcutaneous fat. Hemorrhage is seen in the background along with proliferation of vascular channels. A few reactive germinal centers were present - negative for Desmin, ALK1, AE1/AE3, and ERG, CD34, and CD31. Immunohistochemical stains showed Actin-positive reactive myofibroblasts, S100-positive dendritic reactive histiocytes, CD138 and CD31-positive histiocytes, CD4-positive T lymphocytes and histiocytes, and CD8-positive T cells. Kappa and lambda in-situ hybridization suggested a polytypic population of plasma cells that are CD138 positive. The plasma cells are IgG positive but IgG4 negative. Expert histopathological opinion favoured a reactive inflammatory process, with no convincing evidence of malignancy.

Based on these findings, the patient was commenced on prednisolone 30 mg daily, tapered by 5 mg per week. Propranolol 40 mg twice daily was also prescribed empirically to reduce the vascularity and erythema, though its role in NF remains unclear and is not very well established in the literature. Over the following weeks, the lesion demonstrated gradual regression, and the biopsy site showed progressive healing.

## Discussion

NF can present at any age, but it's more common in adults between 20 and 50 years old. The prevalence is equal in males and females. It can present at any site except the viscera, with the arm and forearm being the most common sites involved, followed by the trunk, then the lower limbs, and lastly the head and neck [2,4,5]. The head and neck are common sites for NF in children; however, it’s rare in adults [6]. The literature describes a reported case of a 64-year-old female with NF on the face, which is similar to our case [7]. In another study, an NF lesion was found in the neck [8]. Most of the lesions of NF reported were solitary, well-defined, soft to firm, measuring 0.5 to 7 cm in maximum diameter, confined to the subcutaneous tissue, with a small number of cases infiltrating into adjacent subcutaneous tissue, muscle, or the parotid gland [5]. On histological grounds, NF could be classified into myxoid, cellular, or fibrous subtypes [9].

Differential diagnosis for NF is extensive; however, it’s essential to differentiate it from malignant lesions in the temple involving deeper structures such as fibromatosis, malignant fibrous histiocytoma, malignant peripheral nerve sheath tumours, and spindle cell sarcomas [10]. The definitive diagnosis is by histopathology, as the radiological findings are nonspecific [5]. The radiological appearance of similar lesions in the head and neck is not widely reported [11]. In a retrospective study of 272 cases of NF found in various parts of the body, ultrasound scans performed on some cases typically showed a well-defined hypoechoic or isoechoic lesion in the subcutaneous tissues [5]. The radiological appearance of a hypoechoic, heterogeneous, poorly defined subcutaneous lesion with increased vascularity in our case is similar to the NF appearance in ultrasound in the literature [12]. The presence of fat necrosis and lymphatic duct dilation in our case may be seen in more infiltrative or reactive cases. Computed tomography imaging in some reported cases revealed low- to iso-dense, well-defined, oval lesions based in the fascia, which is similar to the findings in our case [5]. PET-CT findings of mild FDG uptake in a well-circumscribed soft tissue mass, without evidence of aggressive metabolic activity or distant disease, as in our case, are also typical for NF and help exclude malignancy [13,14].

The diagnosis is established based on the characteristic histological appearance of spindle-shaped to stellate myofibroblasts arranged in a storiform pattern, adjacent to hypocellular myxoid areas, often accompanied by extravasated red blood cells and variable inflammatory cells. The presence of even a moderate amount of plasma cells has been linked to true malignant neoplasm [2,5]. Mitotic figures without atypia are commonly observed. Immunohistochemically, myofibroblasts are positive for Actin, Calponin, CD10, and CD68. Other immunological markers may be used to exclude other differentials [5]. In our case, immunohistochemical stains were positive for Actin and S100. The plasma cells were polytypic with negative IgG4, which supports a benign reactive process.

Management usually involves surgical excision; however, one of the cases reported a rapid resolution after intralesional steroid injection [15]. Spontaneous regression following diagnosis by fine needle aspiration cytology has been reported in some cases [16]. In our case, marked improvement was noted following corticosteroid therapy.

## Conclusions

NF is a rare entity that should be considered as part of the differential diagnosis of benign soft tissue lesions in the head and neck of adults. Proper diagnosis is pivotal, as misdiagnosing NF as a malignant tumour could lead to unnecessary aggressive treatment. The radiological appearance is variable, but most cases reveal hypo to isoechoic lesions in the subcutaneous tissue on ultrasound and well-defined, rounded or oval, low- to iso-dense lesions in the fascia with mild to moderate uptake on PET scan. The definitive diagnosis is made by skin biopsy.
